# Direct Quantification of Heat Generation Due to Inelastic Scattering of Electrons Using a Nanocalorimeter

**DOI:** 10.1002/advs.202002876

**Published:** 2020-12-21

**Authors:** Joonsuk Park, Kiho Bae, Taeho Roy Kim, Christopher Perez, Aditya Sood, Mehdi Asheghi, Kenneth E. Goodson, Woosung Park

**Affiliations:** ^1^ Department of Materials Science and Engineering Stanford University Stanford CA 94305 USA; ^2^ Department of Mechanical Engineering Stanford University Stanford CA 94305 USA; ^3^ Stanford Nano Shared Facilities Stanford University Stanford CA 94305 USA; ^4^ Stanford Institute for Materials and Energy Sciences SLAC National Accelerator Laboratory Menlo Park CA 94025 USA; ^5^ Division of Mechanical Systems Engineering Sookmyung Women's University Seoul 04310 South Korea

**Keywords:** electron beam heating, electron energy loss spectroscopy, heat generation, inelastic scattering, transmission electron microscopy

## Abstract

Transmission electron microscopy (TEM) is arguably the most important tool for atomic‐scale material characterization. A significant portion of the energy of transmitted electrons is transferred to the material under study through inelastic scattering, causing inadvertent damage via ionization, radiolysis, and heating. In particular, heat generation complicates TEM observations as the local temperature can affect material properties. Here, the heat generation due to electron irradiation is quantified using both top‐down and bottom‐up approaches: direct temperature measurements using nanowatt calorimeters as well as the quantification of energy loss due to inelastic scattering events using electron energy loss spectroscopy. Combining both techniques, a microscopic model is developed for beam‐induced heating and to identify the primary electron‐to‐heat conversion mechanism to be associated with valence electrons. Building on these results, the model provides guidelines to estimate temperature rise for general materials with reasonable accuracy. This study extends the ability to quantify thermal impact on materials down to the atomic scale.

## Graphical abstract

Electron beam is converted to heat via inelastic scattering while traversing a solid‐state medium and the generated heat often complicates experimental analysis. A nanocalorimeter enables capturing heat generation induced by electron beams, and in parallel, the electron energy loss spectroscopy measurements reveal that a key energy conversion is to be responsible for the electrons in an outer shell.

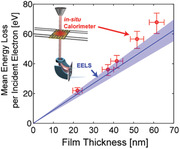

High‐energy electrons are able to traverse thin solid‐state media and probe materials with atomic‐scale resolution.^[^ ^1^, ^2^ ^]^ Insights obtained from transmission electron microscopy (TEM) have led to profound discoveries in materials science, such as defect dynamics,^[^ ^3^, ^4^ ^]^ atomic structure,^[^ ^5^, ^6^ ^]^ and in situ phase formation.^[^ ^7^, ^8^, ^9^, ^10^, ^11^, ^12^ ^]^ The physics of materials is strongly associated with atomic vibrations that manifest as temperature, which is a key element that dictates material characteristics. Although numerous studies have driven the consensus that there exists significant temperature rise due to electron irradiation, local heat generation has rarely been quantified, and thus the energy conversion mechanism has remained elusive.^[^ ^13^, ^14^, ^15^, ^16^, ^17^, ^18^, ^19^, ^20^ ^]^

There have been previous experimental and theoretical efforts to estimate the temperature rise due to electrons incident on a specimen. A primary approach is to solve the heat diffusion equation within a membrane for TEM, the results of which suggest the thermal effects that are independent of membrane thickness.^[^ ^21^ ^]^ To apply this approach to a general specimen however, it is critical to quantify the heat generated from the electron beam.^[^ ^22^ ^]^ In a microscopic model of inelastic scattering, the probability of inelastic scattering is estimated using Bethe‐Bloch theory,^[^ ^23^ ^]^ which is associated with various scattering mechanisms, such as induced radiolysis and knock‐on damage as well as heating.^[^ ^21^ ^]^ It is generally accepted that such inelastic scattering is predominantly associated with valence electrons. Still, there is insufficient understanding about the fraction of electron energy that is converted to thermal energy, as well as predominant energy conversion process.^[^ ^24^ ^]^ Another approach is to directly measure the temperature of the region under investigation. A common way to do this is to identify the temperature dependence in material properties such as diffraction patterns,^[^ ^25^, ^26^, ^27^ ^]^ phase change,^[^ ^28^, ^29^ ^]^ electron energy loss,^[^ ^30^, ^31^ ^]^ and plasmon energy.^[^ ^32^ ^]^ While the experimental methods vary, the accuracy of temperature measurements is generally insufficient for resolving the heat generation due to the electron beam and is limited by the selection of materials whose temperature dependence can be probed by such methods. Thus, it is crucial to build an experimentally validated microscopic model to predict the heat generation for a wide variety of materials.

In this work, we characterize the heat generation due to electron irradiation using both macro‐ and microscopic approaches. Specifically, we 1) measure directly the rate of heat generation under an electron beam using a sensitive nanowatt calorimeter, and 2) quantify the energy conversion per inelastic scattering event using electron energy loss spectroscopy (EELS). A combination of these measurements provides detailed insights into the heat generation process, in particular, identifying the key electron‐thermal energy conversion mechanism, the mean free paths of inelastic scattering, and the associated mean energy loss. As a model specimen, we examine an *α*‐Al_2_O_3_ film deposited via atomic layer deposition (ALD) whose thickness is varied from ≈22 to ≈61 nm. A detailed analysis of the mean energy loss spectrum identifies a key mechanism for the heat generation, which is primarily due to the interaction of electron beam and valence electrons of matter. We apply our findings to general materials as well as conventional TEM configurations in order to estimate the temperature rise under the electron beam spot. This study represents a critical step to methodically describe the temperature rise within TEM to inform future experiments and significantly resolve uncertainty from beam heating.

We develop nanowatt calorimeters that are compatible with commercially available in situ TEM holders as seen in **Figure** **1**a. We note that such calorimeters using resistive thermometry have been demonstrated with various purposes.^[^ ^33^, ^34^, ^35^, ^36^ ^]^ See the Experimental Section for details of the fabrication process. Figure 1b shows a probing area of 13 µm x 13 µm, which is much larger than the beam diameter of ≈7 µm shown in the inset of Figure 1b. We note that the beam size is considerably larger than what is conventionally used for TEM to avoid damage to the material under a strongly focused beam due to effects like ionization. The probing area is surrounded by metal (Cr/Pt) serpentine structures to be used for resistive thermometry and is thermally isolated from a substrate, serving as heat sink, by 500 µm long metal beams. Upon electron beam irradiation in the center of the probing area, we measure the temperature rise using resistive thermometry on the patterned serpentine heaters.^[^ ^34^ ^]^ The measurements are repeated five times in five‐minute intervals to ensure that the high‐energy electron beam causes no significant beam damage or electrical charging of the calorimeter. We control beam current from ≈1 to ≈15.3 nA, corresponding to electron doses of ≈50 to ≈2500 e nm^−2^ s^−1^ at an 80 kV operating voltage. We note that these currents are within the range of typical TEM operation.^[^ ^37^ ^]^ The thermal resistance of the calorimeter is measured to be 1.97 ± 0.05  × 10^−2^ K nW^−1^, which translates nanowatt heat generation into temperature rise (see the Supporting Information).

**Figure 1:**
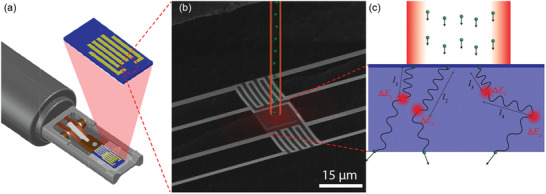
a) Schematic of in‐situ electron microscopy holder and a custom designed nanowatt calorimeter. b) Scanning electron microscope image of a transmission electron microscopy compatible nanowatt calorimeter. The electron beam and heating effects are illustrated on top of the microscopic image at false color. c) A graphical description of inelastic scattering of electrons depicting free‐paths l and energy loss at each inelastic scattering event, Δ*E*.

Along with the thermal measurements, we perform EELS characterization to obtain a microscopic picture of inelastic scattering. As illustrated in Figure 1c, traversing electrons scatter inelastically within a medium with free path *l* and an associated energy loss Δ*E* to the surrounding medium. Collective inelastic scattering is characterized as the mean free path (MFP) *λ*, defined as $1/n\times \sum_{i\;=\;1}^{n}l_{i}$; the MFP is both material and electron energy specific. We specifically investigate the energy loss spectrum for identical samples with the thermal measurements, and the thickness variation from ≈22 to ≈61 nm is used to extract key characteristics of inelastic scattering. See the Experimental Section for details of the EELS measurements.

We compare two measurements on each sample, 1) macroscopic thermal measurements using the calorimeter, and 2) microscopic EELS measurements, as illustrated in **Figure** **2**a. Figure 2b shows the temperature rise due to incident electrons on Al_2_O_3_ while varying the current up to ≈15.3 nA at 80 kV. For the ≈61 nm thick sample, the temperature rise of the entire probing area is estimated to be ≈21 K at ≈15.3 nA. While Joule heating scales proportional to the square of the current, the temperature rise linearly increases with the applied current as seen in Figure 2b. The linearity indicates that individual inelastic scattering events are essentially independent as the amount of heat generated is proportional to the flux of incoming electrons.^[^ ^21^ ^]^ The linearity also suggests that electronic charging effects are negligible from the incident electron beam during the experiments. We note that the measured temperature is the average temperature of the suspended probing area including the serpentine structures for thermometry, so the local temperature under the focused beam could be higher. Using finite element simulations, we estimate that for a 7 µm beam diameter, the temperature under the beam is ≈13% higher than that of the suspended area (see the Supporting Information).

**Figure 2:**
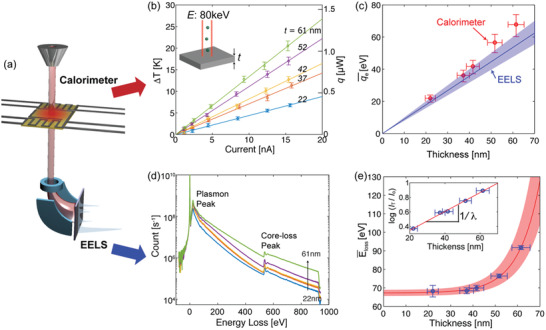
a) A schematic shows simultaneous characterization of temperature rise using the nanocalorimeter and electron energy loss spectroscopy (EELS). The thermal measurements capture the collective heat generated by electron irradiation and the EELS measurements characterize energy loss for individual inelastic scattering events. b) The measured temperature rise with increasing electron beam current at 80 kV accelerating voltage. The film thickness ranges from ≈22 to ≈61 nm, showing increasing temperature rise with increasing its thickness. c) Mean temperature rise per electron incident versus film thickness. The red circles are experimentally measured using the calorimeter, and the blue lines are calculated based on EELS data. The shaded blue region denotes uncertainty in the EELS measurements. d) EELS data with varying film thickness from ≈22 to ≈61 nm. e) Averaged energy loss of the incident beam measured using EELS, which increases with film thickness due to plural scattering. The shaded red region denotes uncertainty in $\bar{E_{\mathrm{loss}}}$, which increases with increasing thickness as the fitting curve becomes more sensitive to the uncertainty in film thickness. The inset shows the log‐ratio measurements used to determine the mean free path. The inverse of the slope is the mean free path due to inelastic scattering.

Using the measured temperature, we evaluate the rate of heat generation^[^ ^33^ ^]^

(1) $$q\;=\frac{\Delta T}{R_{\mathrm{th}}}\;$$

where *q* [W] is heat generation, Δ*T* [K] is the temperature rise, *R*_th_ [K W^−1^] is the thermal resistance of the legs connecting the probing island to the substrate. In the current experiments, the heat generation is estimated to be up to ≈1.1 µW at a beam current of ≈15.3 nA using Equation (1). The applied power ≈1.2 mW, the multiplication of the current and operational voltage 80 kV, is converted to heat generation by a factor of ≈0.1% via inelastic scattering for a ≈61 nm thick sample. The average heat generated due to an individual electron, $\bar{q_{e}}$ is

(2) $$\bar{q_{e}}=\frac{dq}{dn_{e}}\;$$

where *n*_e_ is the number of electrons incident on a sample per second: this is obtained by dividing the beam current by the electron charge. The average heat generation is obtained from the slope of current‐heat generation measurements. We find that the heat generation per incident electron $\bar{q_{e}}$ shows a nearly linear increase with the film thickness as shown in Figure 2c. This observation indicates that the scattering probability increases linearly with increasing film thickness. The measured values deviate slightly from the model prediction, potentially due to either underestimated mean free paths or mean energy losses. We also note that the scattering angle distribution could vary with increasing plural scattering as its thickness increases, leading to another potential source for the deviation.^[^ ^21^ ^]^ Uncertainty analysis is provided in the Supporting Information.

To understand the microscopic picture of heat generation, we perform EELS measurements for the given samples covering both low‐loss and core‐loss regions, as shown Figure 2d. It is clear that the inelastic electron scattering count increases with increasing thickness, indicating plural scattering. As the inelastic collisions are independent, their occurrence obeys Poisson statistics. Considering plural inelastic scattering, the average heat generated due to an incident electron is^[^ ^21^ ^]^

(3) $$\bar{q_{e}}=\sum_{0}^{\infty }\frac{nE_{\mathrm{loss}}}{n!}{\left(\frac{t}{\lambda }\right)}^{n}exp\left(-\frac{t}{\lambda }\right)\;$$

where *t* is the sample thickness, *λ* is the mean free path for inelastic scattering, *n* is the number of inelastic scattering events, and *E*_loss_ is the energy loss per single inelastic scattering. By averaging the energy loss per scattering, the summation can be calculated, and the average heat generated can be expressed as^[^ ^21^ ^]^

(4) $$\bar{q_{e}}=\frac{t}{\lambda }\;\bar{E_{\mathrm{loss}}}$$

where $\bar{E_{\mathrm{loss}}}$ is the mean energy loss per inelastic scattering event. Equation (4) explains the linearly increasing heat generation with increasing film thickness.

To estimate the heat generation per inelastic scattering event, it is required to determine two parameters, the mean free path of inelastic scattering *λ* and the mean energy loss $\bar{E_{\mathrm{loss}}}$. To experimentally determine the electron mean free path *λ* in Al_2_O_3_, we use the log ratio method^[^ ^21^ ^]^ which gives a value of 77.0 ± 7.7 nm for 80 keV electrons, as shown in the inset of Figure 2e. This agrees with literature to within 10%.^[^ ^21^, ^38^ ^]^ See the Supporting Information for the details of the log ratio method.

We further evaluate the mean energy loss per scattering event using EELS data. The measured mean energy loss, including plural scattering, is

(5) $$\bar{E_{\mathrm{loss},\;\exp}}=\int_{0}^{\infty }f\left(E_{\mathrm{loss}}\right)\times E_{\mathrm{loss}}dE_{\mathrm{loss}}\;$$

where *f*(*E*_loss_) is a scattering probability at different energy losses *E*_loss_. We use the EELS distribution to compute the probability *f* (*E*_loss_) =  *I*(*E*_loss_)/*I_T_*, where *I* is the intensity of electrons at energy loss *E*_loss_. *I*_T_ is the total intensity suffering inelastic scattering through the entire energy loss range; this value is calculated using the spectrum after removing the zero‐loss peak. See the Supporting Information for the details of zero‐loss peak removal and associated uncertainty. We note that both $\bar{E_{\mathrm{loss}}}$ and $\bar{E_{\mathrm{loss},\;\exp}}$ represent the averaged value of energy loss among electrons suffering energy loss without those at zero‐loss peak. With increasing film thickness, $\bar{E_{\mathrm{loss},\;\exp}}$ increases exponentially, which indicates that an increasing number of electrons experience plural scattering. As such, to estimate the heat generation using Equation (4), it is important to extract the mean energy loss distribution with a single scattering, $\bar{E_{\mathrm{loss}}}$. To consider the case of predominant single scattering, we fit the data using an exponential function,$\;\;\bar{E_{\mathrm{loss},\exp}}=\;a\cdot exp\left(bt\right)+c$, where *t* is the film thickness. We note that the exponential model is considered to extrapolate the mean energy loss in case of single scattering. When *t*/*λ* is small enough, typically smaller than 0.2, it is reasonable to assume that the transmitted electrons predominantly experience only single scattering.^[^ ^21^ ^]^ We apply a boundary condition of $\frac{\partial \bar{E_{\mathrm{loss},\;\exp}}}{\partial t}{|}_{t\to 0}=\;0$. The y‐intercept of the best fit is the mean energy loss with single scattering, $\bar{E_{\mathrm{loss}}}$, which gives a value of 67.3 ± 1.75 eV.

Given the mean free path and the mean energy loss, we estimate the heat generation using Equation (4) shown in blue solid line with shading in the same color in Figure 2c. The prediction agrees with experimental data to within ≈19%. This agreement suggests that the energy from inelastic scattering is mostly converted to thermal energy, which is then dissipated as heat. We attribute this mismatch between experimental data and the model prediction predominantly to experimental uncertainty, and potentially to also to other energy relaxation processes such as radiolysis.^[^ ^21^ ^]^ We note that X‐ray emission is typically associated with energy loss that is higher than core‐loss since the events are the result of the transition of electrons between shells. As seen in Figure 2d, the relevant count for potential optical loss is an order of magnitude smaller than the others, suggesting that optical loss is not a primary energy loss mechanism.

To understand a key mechanism of heat generation induced by inelastic scattering of electrons, we calculate the cumulative energy loss distribution with experimental electron energy loss spectra. The cumulative function *F* is

(6) $$F\;\left(E_{\mathrm{loss}}\right)=\frac{\int_{0}^{E_{loss}}f\left({E^{\prime }}_{loss}\right){E^{\prime }}_{\mathrm{loss}}d{E^{\prime }}_{\mathrm{loss}}}{\int_{0}^{\infty }f\left({E^{\prime }}_{\mathrm{loss}}\right){E^{\prime }}_{\mathrm{loss}}d{E^{\prime }}_{\mathrm{loss}}}\;$$

We note that $\bar{E_{\mathrm{loss},\;\exp}}=\int_{0}^{\infty }f\left({E^{\prime }}_{\mathrm{loss}}\right)E_{\mathrm{loss}}^{\prime }\;d{E^{\prime }}_{\mathrm{loss}}$. As seen in **Figure** **3**, the accumulation function shows that the energy loss through inelastic scattering is predominantly generated between the plasmon peak and core‐loss peak of the oxygen K shell in Al_2_O_3_. The inelastic scattering between those peaks, ranging from ≈23 to 532 eV, contributes to more than 90% of electron beam heating. We note that this region belongs to the long tail of low‐loss region, which is mainly responsible for the excitation of electrons in the outer shell of atoms in the specimen.^[^ ^9^, ^21^ ^]^ This suggests that thermalization of excited electrons in the outer shell is the prevailing mechanism for electron‐thermal energy conversation. Thus, the mean energy loss $\bar{E_{\mathrm{loss}}}$ is mainly dictated by the number of valence electrons in a specimen, and it is likely to exhibit a periodic behavior with atomic number *Z* as seen in mean free paths.^[^ ^39^ ^]^ As such, we believe that the mean energy loss does not depend on operating voltage, but is rather a material specific property. We also speculate that the variation of the mean energy loss $\bar{E_{\mathrm{loss}}}$ among general materials is within the same order of energy loss. It is known that an average energy loss at 50 keV varies no more than a factor of 3 with atomic number particularly for low atomic number materials (*Z* < 40), where the total cross section ratio between inelastic scattering to elastic is inverse proportional to atomic number.^[^ ^21^ ^]^ We note that additional studies on other materials could help validate our hypotheses on the atomic number dependence of the mean energy loss. The microscopic model suggests that the heat generation can be estimated with mean free paths and mean energy loss. As the scattering probability has been critical for material study, the mean free paths for various materials at different operating voltage are well documented.^[^ ^21^, ^38^, ^40^, ^41^ ^]^ In the case that specimen is thinner than 20% of the mean free path, there is negligible plural inelastic scattering event. As such, the mean energy loss can be estimated using an EELS measurement without much investigation of samples with thickness variation as we have done in this work.

**Figure 3:**
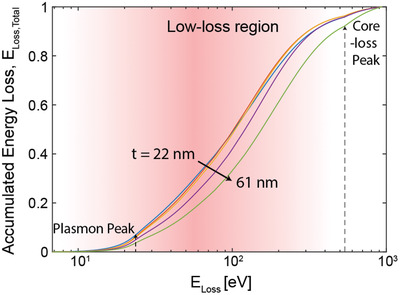
Accumulated electron energy loss with energy loss spectra. Low‐loss region is shaded in red color, which contributes to most of the heat generated upon electron bombardment. The plasmon and core‐loss peaks are marked with dashed arrows on left and right, respectively.

To experimentally validate our model at a different operating voltage, we predict the heat generation per incident electron at 200 kV using material specific mean energy loss. Specifically, we find the mean free paths at 200 kV to be 143 ± 17 nm from literature^[^ ^41^ ^]^ and apply the same mean energy loss of 67.3 ± 1.75 eV, showing agreement with our experimental values within ≈9%. (see the Supporting Information). This agreement also suggests that the heat generation can be estimated solely from EELS measurements with reasonable accuracy. Even without EELS data, $\bar{E_{\mathrm{loss}}}$ can be roughly estimated for general cases within an order of magnitude.

We build a microscopic model for heat generation induced by inelastically scattering electrons in microscopy and extend the model for practical use with general materials. To develop the model, we unambiguously quantify the temperature rise due to an incident electron beam and analyze the associated electron energy loss. By comparing the thermal measurements with EELS measurements, we establish a model to predict heat generation in typical TEM experiments and validate the model experimentally. Based on the EELS data, we identify a critical electron‐thermal energy conversion mechanism, which finds ground to apply the model to general materials with reasonable accuracy and minimal experiments. With a combination of both electron energy loss and mean free paths, we provide a guideline for reasonable heat generation per scattering. Our results make a step towards assessing the specimen temperature rise associated with electron irradiation. This study also addresses the potential thermal complications within electron microscopy and the quantified heat by EELS can be used for a heat source in in situ experiments, thus broadening our understanding of materials physics.

## Experimental Section

##### Fabrication

Al_2_O_3_ was deposited on a 350 µm thick silicon substrate using thermal atomic layer deposition (t‐ALD) at 200 °C with cycles varying from 200 to 600 in 100 cycle increments. The thickness of the alumina films was measured using ellipsometry. On top of the alumina layer, 5 nm of Cr and 40 nm of Pt were deposited and patterned using photolithography and a liftoff process to define the heater and thermometer. The suspended membrane structures were patterned using photolithography and the alumina layer was dry‐etched. The backside of the wafers was coated with aluminum as an etch stop for a deep reactive ion etching process (DRIE). Using DRIE, the substrate was etched through the silicon wafer, leaving few µm of silicon underneath the alumina. The wafers were coated with 3 µm thick photoresist and diced into devices. The photoresist was then removed by soaking the devices in acetone. The remaining silicon below the probing area was removed using a XeF_2_‐based dry etch.

##### Thermal Characterization of Calorimeters

Resistive thermometry of the serpentine patterns was used on the membrane to measure its temperature. The resistance of the serpentine metal lines was measured to be ≈900 Ω, and the temperature coefficient of resistance of the coil was characterized to be 2.007 × 10^−3^ ± 5 × 10^−6^ K^−1^. Since the thermal resistance within the membrane was much smaller than their suspended leg counterparts, the measured temperature was considered to be the average temperature of the membrane. The applied power was calculated to be a sum of the heat generation on the serpentine and half of the power generation on the suspended legs.^[^ ^42^ ^]^ The thermal resistance of the suspended legs were measured to be (1.97 ± 0.05)  × 10^−2^ K nW^−1^. See the Supporting Information for electro‐thermal characterization.

##### Heat Generation Measurements Using a Calorimeter

The nanowatt calorimeter was designed to fit Protochips in‐situ holder and perform the experiments in a FEI Titan TEM at Stanford University. The probing area was surrounded by metal (5 nm thick Cr/40 nm thick Pt) serpentine structures to be used for resistive thermometry and was thermally isolated from a substrate (which serves as a heat sink), by 500 µm long metal beams. The operating voltage was 80 kV and the applied beam current ranges from ≈1 to ≈15.3 nA. The total beam current from the microscope was measured with a phosphor viewing screen with the samples removed from the field of view. Upon electron beam irradiation in the center of the probing area, the temperature rise was measured using resistive thermometry on the patterned serpentine heaters. The measurements were repeated five times in five‐minute intervals to ensure that the high‐energy electron beam causes no significant beam damage or electrical charging of the calorimeter.  It was noted that these currents were within the range of typical TEM operation.^[^ ^37^ ^]^

##### EELS Measurements

The microscope was used for electron energy loss spectroscopy (EELS) measurements. A low accelerating voltage of 80 kV was used because the temperature rise due to the electron beam was the most pronounced. The electron beam in TEM mode was focused to a ≈100 nm diameter on the sample for the EELS measurement using Gatan GIF Quantum. Dual EELS was used to collect both the low loss (0.00001 s exposure time) from −80 to 739.2 eV and the core loss (0.004 s exposure time) from 120 to 939.2 eV with the dispersion of 0.4 eV ch^−1^ and 500 spectrums summed. The EELS data per second exposure was normalized to merge the two energy ranges and performed a zero‐loss peak alignment.

## Conflict of Interest

The authors declare no conflict of interest.

## Supporting information

Supporting Information
