# Supplementary material for: Direct Quantification of Heat Generation Due to Inelastic Scattering of Electrons Using a Nanocalorimeter
Source: Adv Sci (Weinh). 2020 Dec 21;8(3):2002876. doi: 10.1002/advs.202002876 (PMC7856892; doi:10.1002/advs.202002876)
Supplement: Supplementary file 1 — Supporting Information [file ADVS-8-2002876-s001.pdf]

© 2020 Wiley-VCH GmbH

ADVANCED SCIENCE Open Access

Supporting Information

for *Adv. Sci.,* DOI: 10.1002/advs.202002876

Direct quantification of heat generation due to inelastic scattering of electrons using a nano-calorimeter

*Joonsuk Park, Kiho Bae, Taeho Roy Kim, Christopher Perez, Aditya Sood, Mehdi Asheghi, Kenneth E. Goodson, and Woosung Park**

Supporting Information

Direct quantification of heat generation due to inelastic scattering of electrons using a nano-calorimeter

Joonsuk Park, Kiho Bae, Taeho Roy Kim, Christopher Perez, Aditya Sood, Mehdi Asheghi,

*Kenneth E. Goodson, and Woosung Park**

1. Thermal characterization of nanowatt calorimeters

A nanowatt calorimeter is suspended using eight of beams placed on either side symmetrically as shown in **Figure S1**(a). The suspended structure makes the probing area to be thermally isolated. The beams are photolithographically patterned to be identical to each other, and each beam is 500 μm long and 1 μm wide. Figure S1(b) shows a working principle of the calorimeter, where the probing area is isolated by a thermal resistor from its environment. The thermal resistance R_{th} converts the temperature information to corresponding heat generation. To characterize the thermal resistance, we apply a Joule heating on a metal lines using a dc current and measure the temperature of probing area T_H.[1] An electrical resistance of the metal line consists of two parts, a serpentine on the probing island $R_{e,Heater}$ and two connecting beams $2R_{e,Beam}$, and the heated area is in red color in Figure S1 (c). The temperature of probing area T_H is measured using a resistive thermometry, where the electrical resistance is measured using four-probe method. The applied power is calculated to be $Q = Q_{\mathrm{Heater}} + Q_{\mathrm{Beam}}$[1]. The measured thermal resistance is determined to be a derivative of the temperature rise, $\Delta T = T_H - T_0$ over the applied power Q as shown in Figure S1 (d). We note that the thermal characterization is performed in a vacuum chamber and its vacuum level is $\sim 1 \times 10^{-5}$ Torr to minimize convective heat loss. The measured thermal resistance of the calorimeter is $\sim(1.97 \pm 0.05) \times 10^{-2}$ K nW^{-1}, where the uncertainty is statistical uncertainty

among the samples with different thicknesses ranging from ~ 22nm to ~ 61 nm.

To provide a reasonable range of thermal resistance of the beams, we assess the thermal resistance of 8 beams, using thermal resistance analysis. The resistance is $R_{Th} = L/kA$, where L is the length of a beam, k is the thermal conductivity of each material, and A is the cross-section of a beam, the multiplication of thickness t and width w. The resistance of each beam is $R_{Th,Beam} = \left[\left(\frac{L_{Pt}}{k_{Pt}t_{Pt}w}\right)^{-1} + \left(\frac{L_{Cr}}{k_{Cr}t_{Cr}w}\right)^{-1} + \left(\frac{L_{Al2O3}}{k_{Al2O3}t_{Al2O3}w}\right)^{-1}\right]^{-1}$, where the width is 1 μm. The cross-section of a beam is shown in the inset of Figure S1 (d). The total resistance of 8 beams is $R_{Th,Beam}/8$ to be ~ at most $\sim 1.81 \times 10^{-2}$ K nW^{-1} with 61 nm thick Al_2O_3, and the value is calculated using a bulk value for material properties.[2-4] Considering size effects on thin metal films, the thermal resistance value is likely to be suppressed. Thus, the measured thermal resistance value is close to theoretical estimation.

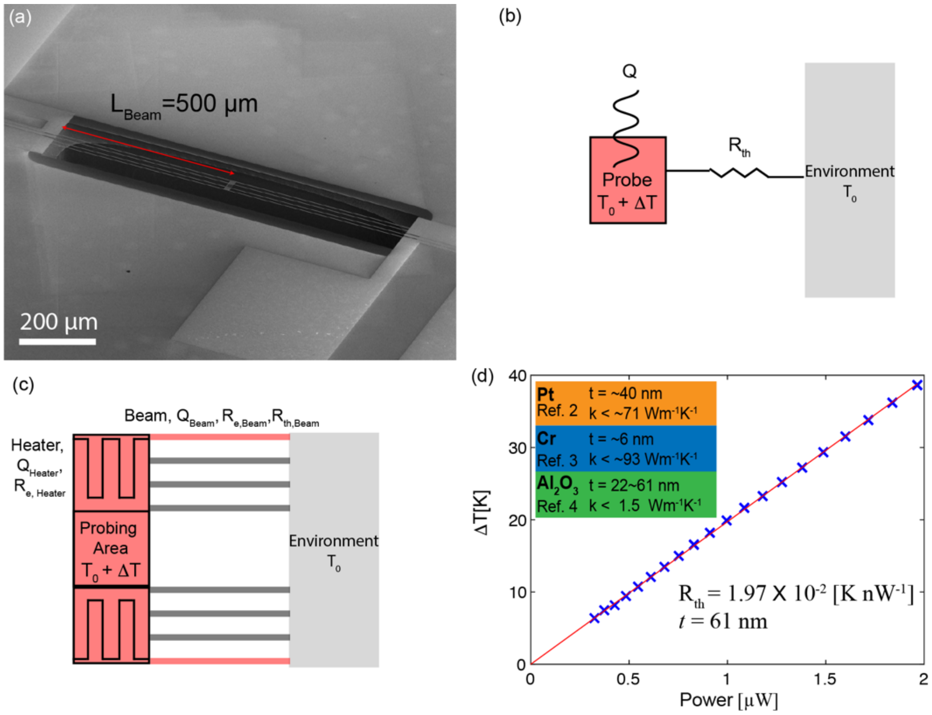

Figure S1. (a) Scanning electron microscopy for the overview of a calorimeter. (b) A schematic showing the working principle of a calorimeter under electron beam irradiation. (c) A schematic showing simplified the structure and associated physical values. (d) Experimentally measured temperature rise of T_H with increasing power, and the temperature is marked in blue cross. The linear best fit is shown in a red solid line. The inset of (d) shows the layered structure of a beam with the thickness of each material.

2. Temperature rise estimation under electron beam

We numerically estimate the temperature rise under electron beam using COMSOL, a commercially available finite element analysis tool. The dimension of the calorimeter is given with identical one with experimental structures as shown in **Figure S2** (a). The thermal conductivity of both Al_2O_3 film and Pt on the island is given to be 1.4 W m^{-1} K^{-1} [4] and 40 W m^{-1} K^{-1} [5]. The suspended leg consists of a metal layer with 40 nm thickness, and its thermal conductivity is given

to be 78 W m^{-1} K^{-1}, which is calculated to have a measured thermal resistance of a calorimeter. We note that the suspended leg actually includes the thermal conductance of Al_2O_3(22~61 nm), Cr (5 nm), and Pt (40 nm). The beam diameter varies from 100 nm to 10 μm, and the applied heat generation is 1 μW. We compute the mean temperature rise ratio of the beam area to the entire of island as described in Figure S2 (b). The temperature under the beam can be ~70 % larger than the average beam temperature. For the 7 μm of beam diameter, the temperature variation is within ~13%. The inset on the right side in Figure S2 (b) shows that the serpentine area is in the same temperature along the metal line.

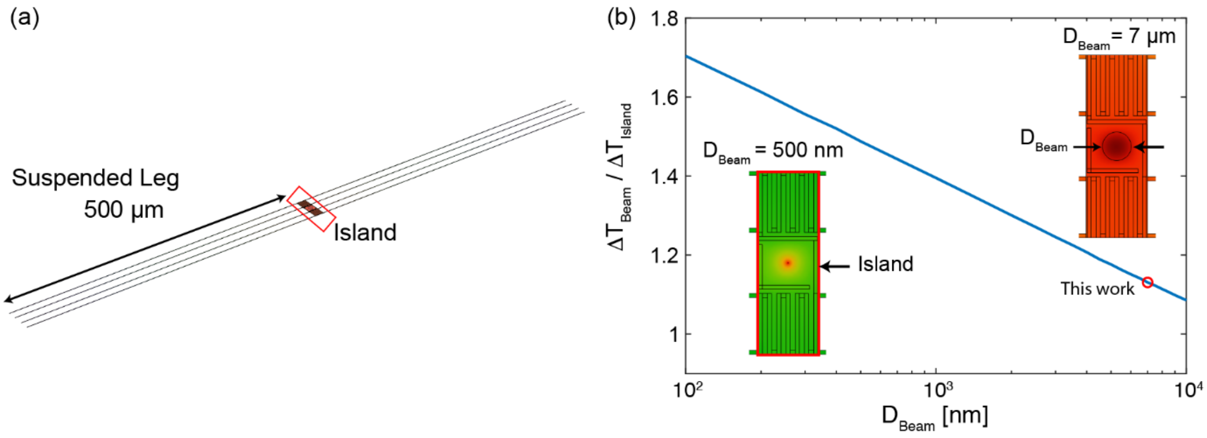

Figure S2. (a) Overview of the calculation structure for numerical simulations. (b) The ratio of temperature rise of the area under beam to that of island. The insets on both left and right show the case with a beam diameter with 500 nm and 7 μm, respectively.

3. Mean free path of inelastic scattering electrons

We use Log-ratio method to determine the mean free path λ of inelastic scattering of electrons based on electron energy loss spectroscopy (EELS).[6] The ratio of specimen thickness t to the mean free path λ is

$$\frac{t}{\lambda} = \log\frac{I_0}{I_T} \tag{S1}$$

where I_T is the total number of electrons contributed in the EELS data and I_0 is the number of electrons in zero loss peak during a given acquisition time. The total number of electrons I_T is obtained by summing the number of counts from -79.4 eV to 939 eV, which can be approximated as the whole spectrum as shown in Figure 2 (d). The electron counts beyond 939 eV is disregarded for its minimal contribution. For I_0, the number of electrons is determined by integrating the electron count from -6.4 eV to 6.4 eV, which is the minimum point near zero loss peak as shown in Figure S1. We note that the minimum intensity energy 6.4 eV is the point where the tails of both zero-loss and neighboring inelastic components approximately cancel out.[6] To estimate the uncertainty associated with the selection of zero loss peak range, we vary the range ± 1 eV as marked as shade in **Figure S3**, and the corresponding uncertainty in MFP is ~ ± 0.1 nm. The most significant uncertainty is attributed to the variation of the mean free path λ depending on the collection angle, which is ~20±10 mrad in this work, and corresponding uncertainty is ~10%. [7,8] Given the uncertainty, the resulting MFP is estimated to be ~77.0 ± 7.7 nm. This value agrees with calculations within ~10% as shown in **Figure S4**.[7] We collect previously reported MFP's for Al_2O_3 up to 200 keV and extrapolate up to 300keV based on computationally calculated values.[9,10]

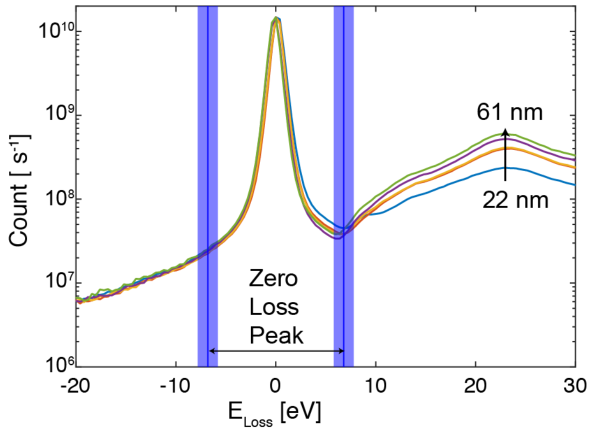

Figure S3. Electron energy loss spectrum near Zero Loss Peak. The blue lines show the range of energy loss to determine the mean free paths of inelastic scattering of electrons, and blue shade shows a considered uncertainty range in energy loss.

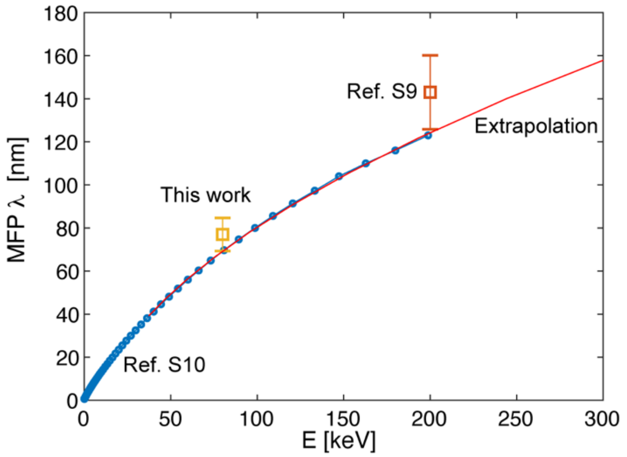

Figure S4. Electron mean free path λ with various electron energy up to 300 keV. The blue dots are obtained from Ref. S10, and lines are connected. The yellow and red hollow squares are the values obtained at 80kV and 200 kV accelerating voltages, respectively. The red solid line is extrapolated based on calculated values in Ref. S10.

4. Zero-loss peak removal and associated uncertainty in Mean Energy Loss

To estimate the mean energy loss for inelastic scattering electrons, we apply three different zero-loss peak removal algorithms: Fit pre-measured zero-loss, Fitted log. tail, and Reflected tail.[11] We note that those algorithms are commercially available. Those methods show the significant difference in electron counts below 7 eV from each other as shown in **Figure S5** (a) while energy loss spectrums are completely overlapped above ~10eV. As such, the uncertainty in $\overline{E_{loss}}$ is predominantly originated from the mismatch in the region with red shade in Figure S5 (a). The error propagates to the evaluation of $\overline{E_{loss}}$, and the mismatch decreases with increasing thickness as shown in Figure S5. (b). We set the uncertainty of $\overline{E_{loss}}$ to be the maximum difference among dissimilar zero-loss peak removal methods. The resulting $\overline{E_{loss}}$ is estimated to be ~ 67.3 ± 1.75 eV.

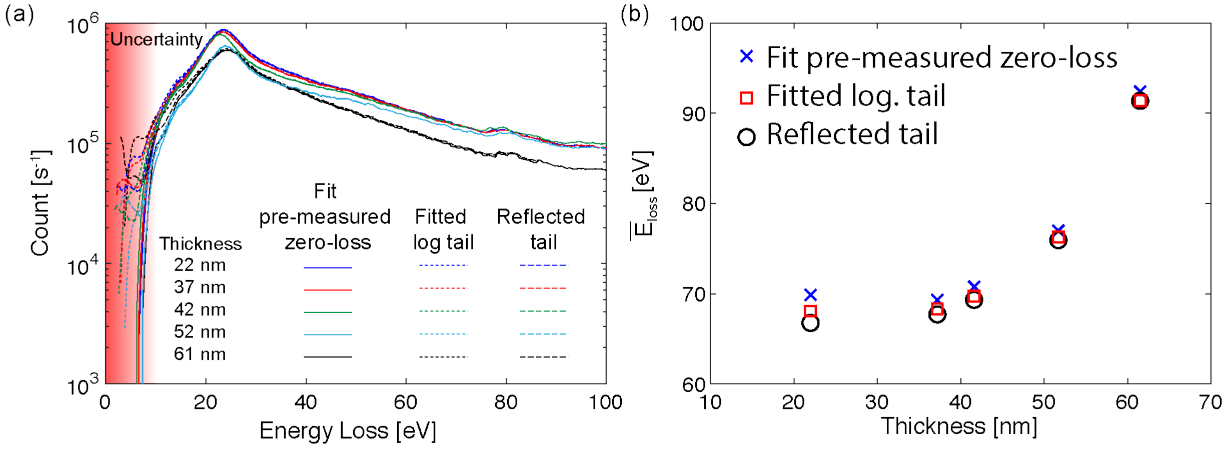

Figure S5 (a) Energy loss spectrum removed zero-loss peak with three methods: Fit pre-measured zero-loss, Fitted log. tail, and Reflected tail, corresponding to solid, dotted, and dashed lines, respectively. The red shaded area shows predominant mismatch in electron counts. (b) The estimated mean energy loss depending on zero-loss removal algorithms above mentioned.

5. Heat generation at 200kV and 300kV

We present the measured heat generation per incident electron at 200kV and 300kV as well as predict the heat generation with the incident beam using a model that is developed in this work.

We note that the experimental data is obtained using the same set of samples at 80 kV and share the material information. We recast the equation of heat generation per incident electron to be

$$\overline{q_e} = \frac{t}{\lambda}\overline{E_{loss}} \tag{S2}$$

We obtain the mean free path at 200kV from literature, and the value is ~ 143 ± 17 nm[9]. For 300kV, we choose 150 nm, 160 nm, and 170 nm to provide guidance since the experimentally measured value is unavailable. We note that the extrapolated mean free paths based on calculations is ~158 nm at 300kV.[10] The mean energy loss from Eq. (6), and the value is ~ 67.3 ± 1.75 eV. For 200kV, the model prediction agrees with experimental data within ~8.8 % as shown in Figure S6 (a). This indicates that the mean energy loss is consistent with electron voltage while its scattering probability depends on the kinetic energy of electrons.

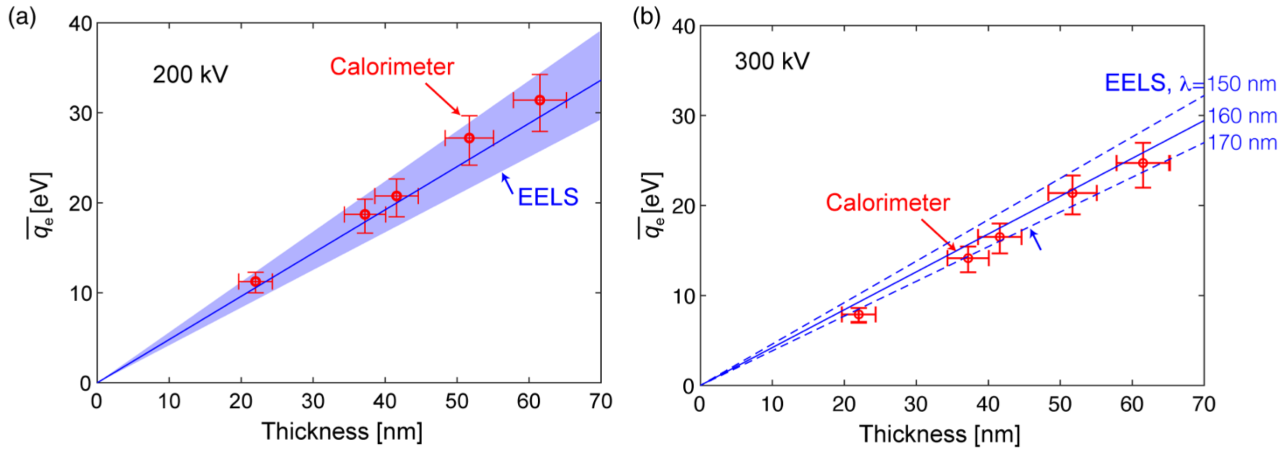

Figure S6. Heat generation per incident electrons with varying film thicknss up to 70 nm at (a) 200 kV and (b) 300kV. The red circles are the measured values. In (a), the blue solid line is the prediction from a microscopic model and the blue shaded region shows the uncertainty from electron energy loss spectroscopy. In (b), the blue prediction lines corresponds to mean free paths of 150 nm, 160 nm, and 170 nm as noted.

6. Uncertainty Analysis for calorimeters

6.1. Uncertainty in sample thickness

The uncertainty in film thickness is mainly contributed to two components: 1) non-uniformity in atomic layer deposition (ALD) films and 2) a native silicon dioxide on silicon wafers. To quantify the non-uniformity of film thickness, we measure the thickness of samples across a wafer using an ellipsometer. For all processed wafers, the maximum thickness variation is evaluated to be ~4%, which is consistent in the wafers with cycles from 200 to 600. We note that the presence of native oxide on silicon wafers potentially increase the alumina film thickness. The contribution of native oxide is estimated to be up to ~1.6 nm.[12]

6.2. Uncertainty in temperature measurements

We use a resistive thermometry to measure the temperature of probing area. The resistance of a metal line is

$$R = R_0(1 + \alpha \Delta T) \quad \text{(S3)}$$

where R_0 is an electrical resistance at room temperature, α is a temperature coefficient of resistance (TCR) of a Pt line, and ΔT is the temperature rise due to electron bombardment with respect to the room temperature. As the uncertainty of electrical resistance is negligible, TCR is the most significant source of uncertainty in temperature measurements. We selectively characterize three samples from the samples with all the thickness, and the mean variation of TCR among samples is ~5%.

7. Temperature Estimation in a Conventional TEM Specimen

We estimate the temperature rise of a specimen under parallel electron beam in a conventional TEM scheme, a particle on a film. Typically, a nanoparticle is positioned on a thin film of lacey carbon, SiN_x, or SiO_x. A typical structure is modeled as a cylindrical nanoparticle as shown in the left inset of Figure S7. The heat generated within the particle and film is

$$q_{particle} = n_{e,particle} \times \frac{t_{particle}}{\lambda} \times \overline{E_{loss}} \tag{S4}$$

$$q_{film} = n_{e,film} \times \frac{t_{film}}{\lambda} \times \overline{E_{loss}} \tag{S5}$$

where subscript both *particle* and *film* indicate an associated quantity for a particle and a film, respectively. n_e is the number of electrons incident per second, calculated by multiplying electron dose with the corresponding area. t is a sample thickness where electrons are transmitting. We use both the mean free path λ and the mean energy loss $\overline{E_{loss}}$ for Al_2O_3 by assuming that these values are similar for other materials to first order. The TEM sample structure can be modeled using a thermal resistance network as shown in the right inset of **Figure S7**. Given structure can be modeled using thermal resistances network, comprising mainly two parts: 1) interfacial resistance between the sample and the supporting film $R_{Interface}$ and 2) the spreading resistance from the beam to substrate heat sink R_{Film}. The resistances are

$$R_{Interface} = TBR'' \times \frac{4}{\pi {D_{particle}}^2} \tag{S6}$$

$$R_{Film} = \frac{log\left(D_{film}/D_{beam}\right)}{2\pi t_{film} k_{film}} \tag{S7}$$

where TBR'' is the thermal boundary resistance between the sample and the film. t_{film} and k_{film} are the thickness and the thermal conductivity of the film, respectively. D_{film} is 100 µm for the size of

a single cell of a TEM grid. The maximum temperature rise is found at the particle under the beam, and the value is calculated using

$$\Delta T_{particle} = q_{particle} \times (R_{Interface} + R_{Film}) + q_{film} \times R_{Film} \quad \text{(S8)}$$

Typical values are as follows: TBR ranges from 10 to 1000 m^2 K GW^{-1} [13], the thermal conductivity k_{film} and the thickness are assumed to be ~0.15 W m^{-1} K^{-1} [14] and 10 nm for a lacey carbon, respectively. D_{film} is approximated to be 100 μm, a common size of a TEM grid. We choose D_{sample} = 10 nm to render a particle-like structure, and beam diameters D_{Beam} of 1, 2, and 5 μm are used. We vary the electron beam dose from 100 to 10000 e nm^{-2} s^{-1} and estimate the temperature as shown in Figure S7.

Calculated by the above thermal model, the heat dissipation is primarily generated and limited by the thermal resistance of the supporting films. We note that the heat generation due to a particle is insignificant in this calculation as the heat generation is proportional to the area exposed to an electron beam. While the ratio of R_{Film} to $R_{Interface}$ is smaller than 5% at most, the heat generation on a particle is negligible, less than 0.1% of the counterpart on a film. Given calculations, we estimate the maximum temperature rise to be close to 100 K solely due to the electron beam incident on TEM samples. Our calculations suggest that both heat generation and dissipation is limited by the supporting films.

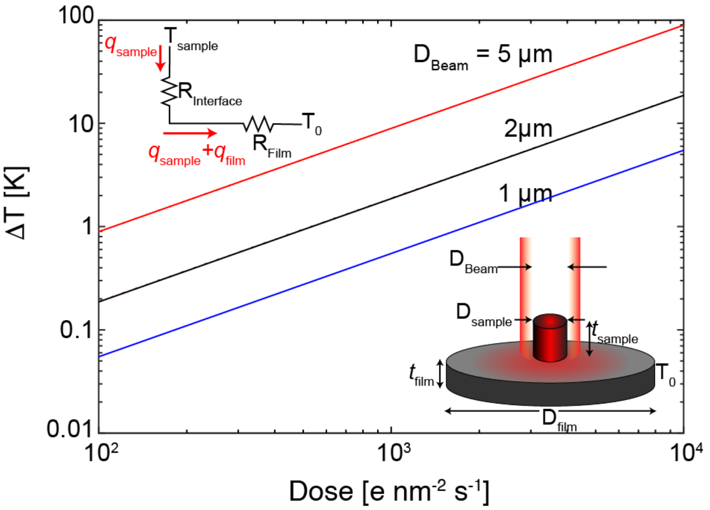

Figure S7. Calculated temperature rise upon electron bombardment with varying electron beam size and electron beam dose. For this calculation, the film thickness of t_{film} = 10 nm. The inset on the lower right corner illustrates a schematic of a conventional sample structure in electron microscopy. The inset on the upper left shows a thermal resistance network of the specimen.

References

[1] L. Shi, D. LI, C. Yu, W. Jang, D. Kim, Z. Yao, P. Kim, A. Majumdar, *J. Heat Transfer* **2003**,*125*, 881.

[2] M. Laubitz, M. v. d. Meer, *Can. J. Phys.* **1966**,*44*, 3173.

[3] J. Moore, R. Williams, R. Graves, *J. Appl. Phys.* **1977**,*48*, 610.

[4] C. S. Gorham, J. T. Gaskins, G. N. Parsons, M. D. Losego, P. E. Hopkins, *Appl. Phys. Lett.* **2014**,*104*, 253107.

[5] S. Yoneoka, J. Lee, M. Liger, G. Yama, T. Kodama, M. Gunji, J. Provine, R. T. Howe, K. E. Goodson, T. W. Kenny, *Nano Lett.* **2012**,*12*, 683.

[6] R. F. Egerton, *Electron Energy-loss Spectroscopy in the Electron Microscope*, Springer Science & Business Media, Berlin **2011**.

[7] Y.-Y. Yang, R. Egerton, *Micron* **1995**,*26*, 1.

[8] H. Meltzman, Y. Kauffmann, P. Thangadurai, M. Drozdov, M. Baram, D. Brandon, W. Kaplan, *J. Microsc.* **2009**,*236*, 165.

[9] K. Iakoubovskii, K. Mitsuishi, Y. Nakayama, K. Furuya, *Phys. Rev. B* **2008**,*77*, 104102.

[10] H. Shinotsuka, S. Tanuma, C. J. Powell, D. R. Penn, *Surf. Interface Anal.* **2019**,*51*, 427.

[11] E. DigitalMicrograph, Gatan Inc., Pleasanton, 2003.

[12] M. Morita, T. Ohmi, E. Hasegawa, M. Kawakami, M. Ohwada, *J. Appl. Phys.* **1990**,*68*, 1272.

[13] F. Incropera, D. Dewitt, *Introduction to heat transfer*, John Wiley and Sons Inc, United States **1985**.

[14] A. A. Balandin, M. Shamsa, W. L. Liu, C. Casiraghi, A. C. Ferrari, *Appl. Phys. Lett.* **2008**,*93*, 043115.
